# Common variable immunodeficiency misdiagnosed as Crohn Disease

**DOI:** 10.1186/1939-4551-8-S1-A267

**Published:** 2015-04-08

**Authors:** Daniella Moore, Fabiane Dias, Eliane Esberard, Jorge Mugayar, Marcia Costa, Simone Pestana, Jose Laerte Boechat, Rossana Rabelo, Amanda Seba

**Affiliations:** 1 Universidade Federal Fluminense, Brazil; 2 Hospital Universitário Antonio Pedro, Brazil; 3 Clementino Fraga Filho Hospital, Federal University of Rio De Janeiro, Brazil

## Background

Common variable immunodeficiency (CVID) is one of the most common primary immunodeficiency in adults and is characterized by defective antibody production, low levels of serum immunoglobulins and increased susceptibility to infection. About 20% of developed different gastrointestinal pathology. But also the pathology of the gastrointestinal tract in patients with CVID showed a wide spectrum of histological patterns which could mimic many conditions such as inflammatory bowel disease.

## Objective

The presented case highlights the importance of the evaluation of primary immunodeficiency in patients with chronic gastrointestinal disorders.

## Case report

JRLM, 38 years, male, natural from Rio de Janeiro, Brazil, with a history of recurrent respiratory infections during childhood, begins to presented diarrhea (3-4 episodes of loose stools /day) in 2004 without mucus or blood. The colonoscopy showed moderate erosive pancolitis and a nodular ileitis. It was therefore decided to start treatment with mesalazine. In 2005 the colonoscopy was repeated and the patient received the diagnosis of Crohn´s Disease and mesalazine was replaced by azathioprine, however the control of diarrhea was not obtained. In 2013 the patient begun treatment in Antonio Pedro Hospital where the colonoscopy was repeated and showed inespecific pancolitis and the endoscopic aspect was not suggestive of Crohn´s disease. The stool examination for parasites showed the presence of Giardia lamblia and Blastocystis hominis and the treatment with metronidazole and ivermectin control the diarrhea. In that time, screening tests for serum immunoglobulin were requested and showed IgG=212mg/dl (751-1555), IgA= <6,67mg/dl (82-453) and IgM=10.2mg/dl (46-304). The patient was diagnosed with common variable immunodeficiency and started treatment with prophylactic amoxicillin and intravenous immunoglobulin with remarkable improvement.

## Discussion

Several studies have documented increased incidence of inflammatory bowel disease in CVID patients. Some patients require in addition to intravenous immunoglobulin replacement therapy, steroids or immunosuppressive drugs to control diarrhea. However our patient showed improvement only with the prophylactic amoxicillin and intravenous immunoglobulin therapy. The chronic diarrhea and the colonoscopy findings mimic the presence of inflammatory bowel disease and mislead the physician for a period and lead to the exclusive treatment of inflammatory bowel disease.

## Consent

Written informed consent was obtained from the patient for publication of this abstract and any accompanying images. A copy of the written consent is available for review by the Editor of this journal.

